# Kepler Algorithm for Large-Scale Systems of Economic Dispatch with Heat Optimization

**DOI:** 10.3390/biomimetics8080608

**Published:** 2023-12-14

**Authors:** Sultan Hassan Hakmi, Abdullah M. Shaheen, Hashim Alnami, Ghareeb Moustafa, Ahmed Ginidi

**Affiliations:** 1 Electrical Engineering Department, College of Engineering, Jazan University, Jazan 45142, Saudi Arabia; shhakmi@jazanu.edu.sa (S.H.H.); halnami@jazanu.edu.sa (H.A.); 2 Department of Electrical Engineering, Faculty of Engineering, Suez University, Suez P.O. Box 43221, Egypt; ahmed.ginidi@eng.suezuni.edu.eg

**Keywords:** Kepler optimization algorithm, economic dispatch, valve point loading effect, large 192-unit system, CHPUED

## Abstract

Combined Heat and Power Units Economic Dispatch (CHPUED) is a challenging non-convex optimization challenge in the power system that aims at decreasing the production cost by scheduling the heat and power generation outputs to dedicated units. In this article, a Kepler optimization algorithm (KOA) is designed and employed to handle the CHPUED issue under valve points impacts in large-scale systems. The proposed KOA is used to forecast the position and motion of planets at any given time based on Kepler’s principles of planetary motion. The large 48-unit, 96-unit, and 192-unit systems are considered in this study to manifest the superiority of the developed KOA, which reduces the fuel costs to 116,650.0870 USD/h, 234,285.2584 USD/h, and 487,145.2000 USD/h, respectively. Moreover, the dwarf mongoose optimization algorithm (DMOA), the energy valley optimizer (EVO), gray wolf optimization (GWO), and particle swarm optimization (PSO) are studied in this article in a comparative manner with the KOA when considering the 192-unit test system. For this large-scale system, the presented KOA successfully achieves improvements of 19.43%, 17.49%, 39.19%, and 62.83% compared to the DMOA, the EVO, GWO, and PSO, respectively. Furthermore, a feasibility study is conducted for the 192-unit test system, which demonstrates the superiority and robustness of the proposed KOA in obtaining all operating points between the boundaries without any violations.

## 1. Introduction

### 1.1. Motivation of the Study

Power and heat systems that are combined bear the responsibility of meeting both electrical and heating demands, which contradicts the conventional methods of electricity generation. As a result, the goal of the combined heat and power economic dispatch issue is to minimize both the total cost of producing electrical power and heat under certain operational restrictions, including the production attributes of combined power and heat (CHP) units, the electrical power, the heat balance, the production capacities of heat-only and power-only units, etc. [1]. Formally, the CHPUED problem under consideration can be represented mathematically as a non-convex constrained optimization issue. By using appropriate optimization techniques with the CHPUED issue, the ideal generation schedule for power and heat can be established. One of the primary motivations for this study is the increasing demand for efficient and sustainable power generation in large-scale systems. Also, the researchers recognize the need for advanced optimization algorithms that can handle the challenges posed by large-scale systems and can incorporate heat optimization aspects. Moreover, the inclusion of heat optimization in the study’s objectives highlights another important motivation. Heat optimization involves optimizing the utilization of waste heat generated during power generation processes, thereby enhancing the overall energy efficiency.

### 1.2. Literature Review

Economic load dispatch (ELD) is a crucial optimization problem in power systems that aims to allocate the optimal power generation among multiple generating units to meet the electricity demand at minimum cost while satisfying various operational constraints [2]. The objective of ELD is to determine the optimal power output for each generator, taking into account factors such as fuel cost, transmission losses, and system constraints. The goal is to minimize the total cost of generating electricity while ensuring that the power supply remains reliable and stable [3]. ELD considers various parameters, including the cost curves of generators, generation limits, ramp rates, transmission constraints, and renewable energies [4]. In Ref. [5], the application of an enhanced particle swarm optimizer was described for a dispatching model that takes into account various factors. These include market power sales benefits, environmental benefits from grid-connected operations, and system operation and maintenance costs. The objective of the model was to optimize the benefits of a combined system consisting of wind, photovoltaic, and concentrating solar power by optimizing its grid-connected performance. In Ref. [6], the sparrow search algorithm was utilized to address the economic load dispatch (ELD) problem in renewable integrated microgrids. The goal was to determine the optimal power output of all distributed energy sources within the microgrid, considering renewable sources, to meet the load demand at the lowest possible cost.

The initial attempts at addressing the CHPUED problem in the literature involved using deterministic methods, which required investigating the solution space over a limited number of repetitions, with the aid of a set of deterministic bounding procedures. The CHPUED problem is divided into two subproblems as indicated in Ref. [1], with the power and heat dispatches being handled separately by the Lagrangian relaxation approach. In Ref. [5], a bi-level structure with lower and upper levels was used to tackle the CHPUED issue. Additionally, in this study, the global limitations were managed at the top level, and unit generation was achieved at the lower level. Another study [6] used Benders decomposition to divide the CHPUED problem into a master problem and a sub-problem. In each iteration, the master issue was first solved to determine the heat productions, and the subproblem was then solved to produce the best power generation. Power dispatch and unit commitment issues combine to form CHPUED [7]. Thus, inner and outer Bender decompositions, in the two Benders decomposition techniques, were proposed. While the outer inner problem addressed a power dispatch, the outer master problem resolved the unit commitment problem by identifying the on/off status of each unit. Moreover, the inner decomposition ensured constraint fulfillment. Other traditional methods used to address the CHPUED problem in the literature included a branch-and-bound algorithm [8] and mixed-integer nonlinear programming [9].

The CHPUED problem becomes a non-convex problem when it is defined within the constraints of the valve point effect. Although the optimal solution is guaranteed by traditional approaches, these might not be able to solve the non-convex CHPUED issue. Metaheuristics carry out certain procedures that use their stochastic nature to investigate the solution space. Although they cannot guarantee that they will be close to the global optimum, they can nevertheless handle non-differentiable and non-convex problems and are simple to employ [10,11,12]. Harmony search (HS), differential evolution (DE), particle swarm optimization (PSO), and the genetic algorithm (GA) are some well-known examples of metaheuristics algorithms. A self-adaptive real-coded genetic algorithm was used to solve the non-convex CHPUED problem in Ref. [13]. In Ref. [14], PSO was utilized to solve the CHPUED problem in order to minimize the total generation cost. However, the application of PSO in this context has been limited to a small-scale system consisting of only six units, comprising two generators, two heat-only units, and two power-only units. Furthermore, the issue of transmission losses has not been considered, leading to certain inconsistencies. Additionally, the validation of the PSO approach has not been thoroughly discussed. The research conducted by [15] presented a novel approach to address the CHPUED problem by introducing a modified version of the bat optimization algorithm. This modified algorithm was designed to handle three distinct types of units to minimize the operating costs. In [10], an elitist variant of the cuckoo search algorithm has been applied for benchmark functions. In addition to this application, the elitist cuckoo search has been dedicated to solving the CHPUED problem. On the other side, the computational burden of 1000 iterations for the five- and seven-unit systems is considered, which represents more than three times that of other methods [16] with 300 iterations. Additionally, a new approach to constraint control with penalties is shown. Considering several cases, the proposed method’s effectiveness is tested. In Ref. [17], a novel mutation technique known as the Mühlenbein mutation is presented to increase convergence and solution time. Real-coded GA is used to solve the CHPUED problem while taking into account the transmission system losses and the valve point impact caused by the thermal units. In Ref. [18], Cauchy distribution is used to increase the PSO’s efficiency in addressing the CHPUED problem. Six separate test systems are used to validate the proposed method. Another population-based metaheuristic, PSO with time-varying acceleration coefficients, is utilized in Ref. [19] to solve the CHPUED issue, which includes generating constraints. In [20], the CHPUED problem has been treated, including renewable wind energy in order to overcome the intermittent renewable and load variations. In this study, it was solved by the metaphor-less Rao-3 algorithm, which involves the reliance on metaphors to enhance transparency, simplicity, and ease of understanding.

Along with these traditional stochastic optimization techniques, new metaheuristics have also recently been used to tackle the CHPUED problem, which represents the subject of this study. In [21], a non-convex CHPUED issue has been solved using a heap technique, which is then tested on four separate case studies with 7, 24, 48, and 96 generating units. However, the large 192-unit system has not been accounted for in this study. CHPUED, as a non-convex optimization problem, is formulated in this manuscript and solved via the Kepler optimization algorithm (KOA). In 2015 [22], an attempt to simulate the KOA in a hybrid algorithm with the gravitational search algorithm (GSA) was introduced for solving numerical benchmarks. This hybrid algorithm made use of only the first Kepler’s law with high simplification. It mimicked his law by focusing the search on the sun position by either multiplying it by a uniformly distributed pseudo-random number or by adding a distance component between the sun position and each solution. In the presented study, the KOA is developed in a complete optimization framework simulating different laws and features regarding Kepler’s concept [23]. The KOA enables a more efficient exploitation and exploration of the search space due to the candidate solutions’ (planets’) varying distances from the sun at various times. Each planet in the KOA represents a candidate solution with regard to the optimal solution (the sun), which is changed at random during the optimization process. All the practical constraints of CHPUED are considered in this paper. All operating points of different units are obtained between the boundaries without any violations.

### 1.3. Paper Contributions

The main points of this manuscript are summarized as follows:

- A KOA is developed and applied for the non-convex CHPUED.
- Three large-scale systems of 48, 96, and 192 units are considered tests for evaluating the effectiveness of the KOA.
- To assess the efficacy of the applied KOA, recent optimization algorithms are employed.
- To estimate the KOA’s superiority, comparisons are illustrated with various well-known methodologies that have been presented in the scientific literature.
- To demonstrate the accuracy of the proposed KOA, a feasibility study is investigated.

### 1.4. Paper Organization

The content of the paper is structured into five sections. The KOA algorithm is introduced in Section 2. The CHPUED optimization model is formulated in Section 3. Section 4 contains a detailed analysis and discussion of the numerical results for the three case studies. The final conclusions are drawn in Section 5.

## 2. CHPUED Formulation

### 2.1. Objective

**Figure 1:**
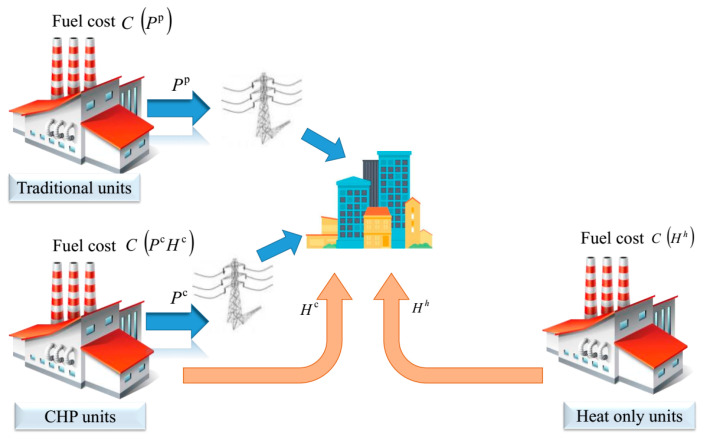
Graphic representation of economic CHPUED issue.

The major objective of the CHPUED challenge is to reduce the fuel expenses associated with producing heat and electricity. As a result, CHPUED is described as an objective function that is subjected to a number of restrictions. Figure 1 displays a graphic representation of the economic CHPUED issue including their participants.

As the major objective of CHPUED is to lessen the whole cost of heat and power production, the whole cost target function (*WCTF*) can be described as the whole cost of the power-only, power–heat amalgamation, and heat-only units [24] as depicted in the following equation:

(1) $$\mathrm{Min}\;WCTF=\sum_{i=1}^{N_{pr}}\;CT1_{i}(PR_{i}^{pr})+\sum_{j=1}^{N_{ht}}CT2_{j}(HT_{j}^{ht})+\sum_{k=1}^{N_{chp}}CT3_{k}(PR_{k}^{chp},HT_{K}^{chp})\;(\$/h)$$

where the terms $CT1_{i}(PR_{i}^{pr})$ and $CT2_{j}(HT_{j}^{ht})$ demonstrate the *i*^th^ power-only and the *j*^th^ heat-only units’ cost, respectively, whilst the term $CT3_{k}(PR_{k}^{chp},HT_{k}^{chp})$ determines the *k*^th^ CHP. The symbols *N_ht_* and *N_pr_* depict the heat-only and power-only units’ number, respectively, while *N_chp_* illustrates the CHP units’ number.

The mathematical representation of three cost target functions $CT1_{i}(PR_{i}^{pr})$, $CT2_{j}(HT_{j}^{ht})$, and $CT3_{k}(PR_{k}^{chp},HT_{k}^{chp})$ can be described as follows:

(1) *CT*1*_i_* of *i*^th^ power units
  (2) $$CT1_{i}(PR_{i}^{pr})=\delta 1_{i}{\left(PR_{i}^{pr}\right)}^{2}+\delta 2_{i}PR_{i}^{p}+\delta 3_{i}+\left|\lambda_{i}\sin(\rho_{i}(PR_{i}^{pr_{\min}}-PR_{i}^{pr}))\right|\;(\$/h)$$
(2) *CT*2*_j_* of *j*^th^ heat units
  (3) $$CT2_{j}(HT_{j}^{ht})=\gamma 1_{j}{\left(HT_{j}^{ht}\right)}^{2}+\gamma 2_{j}HT_{j}^{ht}+\gamma 3_{j}\;(\$/h)$$
(3) *CT*3*_k_* of *k*^th^ CHP units
  (4) $$CT3_{k}(PR_{k}^{chp},HT_{k}^{chp})=\alpha 1_{k}{\left(PR_{k}^{chp}\right)}^{2}+\alpha 2_{k}PR_{k}^{chp}+\alpha 3_{k}+\alpha 4_{k}{\left(HT_{k}^{chp}\right)}^{2}+\alpha 5_{k}HT_{k}^{chp}+\alpha 6_{k}HT_{k}^{chp}PR_{k}^{chp}\;(\$/h)$$
  where the elements (*δ*1*_i_*, *δ*2*_i_*, and *δ*3*_i_*) and (*γ*1*_j_*, *γ*2*_j_*, and *γ*3*_j_*) describe the *i*^th^ power-only and the *j*^th^ heat-only units’ cost coefficients, while the elements ($\alpha 1_{k},\alpha 2_{k},\alpha 3_{k},\alpha 4_{k},\alpha 5_{k}$ and $\alpha 6_{k}$) represent the *k*^th^ CHP unit’s cost coefficients. The non-differentiability and non-convexity of CHPUED, which signify the valve-point impacts, are determined by sinusoidal terms, as explained in Equation (2) [25,26].

### 2.2. Constraints

The following constraints are taken into account when minimizing the specified objective function. The balance among power generation and demand can be calculated using Equation (5):

(1) Power balance constraint
  (5) $$\sum_{i=1}^{N_{pr}}PR_{i}^{pr}+\sum_{k=1}^{N_{chp}}PR_{k}^{chp}=PR_{demand}$$
  where $PR_{demand}$ explains the power demand.
(2) Limits of power units’ capacity
  (6) $$PR_{i}^{pr_{\min}}\leq PR_{i}^{pr}\leq PR_{i}^{pr_{\max}}\;i=1,\ldots ,N_{pr},$$
(3) Heat balance constraint
  (7) $$\sum_{i=1}^{N_{chp}}HT_{i}^{chp}+\sum_{j=1}^{N_{ht}}HT_{j}^{ht}=HT_{demand},$$
  where $H_{demand}$ proves thermal demand.
(4) Heat units’ generation limits
  (8) $$HT_{j}^{ht_{\min}}\leq HT_{j}^{ht}\leq HT_{j}^{ht_{\max}}\;j=1,\ldots ,N_{ht},$$
(5) CHP capacity limits
  (9) $$PR_{i}^{chp_{\min}}(HT_{i}^{chp})\leq PR_{i}^{chp}\leq PR_{i}^{chp_{\max}}(HT_{i}^{chp})\;i=1,\ldots ,N_{chp},$$
  (10) $$HT_{i}^{chp_{\min}}(PR_{i}^{chp})\leq HT_{i}^{chp_{}}\leq HT_{i}^{chp_{\max}}(PR_{i}^{chp})\;i=1,\ldots ,N_{chp},$$
  where the heat and power unit boundaries are demonstrated by the superscripts “min” and “max”.

## 3. Mathematical Model of KOA

The Kepler optimization algorithm (KOA), proposed in [23], is inspired from Kepler’s laws for planetary motion. Each planet in the KOA acts as a candidate solution and can be updated at random during the optimization process in relation to the best-possible solution (the sun). A set of initial objects (possible solutions) containing stochastic orbitals is used by the KOA to begin the search process. During this phase, each object is first placed in an orbit at a random location. The KOA runs in iterations after assessing the original set’s fitness until the termination condition is satisfied. Because iteration is a term that is frequently used in solar system theory and cosmic cosmology, the term “time” is used in the present study instead of the iteration. In the following section, the KOA will be presented in six steps.

### 3.1. Step 1: Initialization Process

In this procedure, the decision parameters of an optimization issue, which are represented by a number of planets *N_p_* and called the population size, will be distributed at random in *Dim* dimensions as follows:

(11) $${\vec{X}}_{i,j}(0)=r_{1}\times {\vec{X}}_{j,up}+{\vec{X}}_{j,low}(1-r_{1}),i=1:N_{p};j=1:Dim$$

where *X_i,j_* signifies the *i*^th^ candidate solution (planet); *N_p_* represents the number of candidate solutions in the search space; *X*_*j*,*low*_ and *X*_*j*,*up*_ characterize the lower and upper limits of the *j*^th^ decision parameter, respectively; *Dim* denotes the issue dimension to be enhanced; and *r* stands for a number randomly generated between 0 and 1.

### 3.2. Step 2: Calculating an Object’s Velocity

An object’s velocity is influenced by $V_{{}_{i}}(t)$ where it is in relation to the sun. In other words, a planet moves faster when it is near to the sun and slower when it is farther away. The sun’s gravity is quite powerful when an object is close to it, so the planet tries to move faster to escape being drawn toward the sun. The weak gravity of the sun will force an object’s velocity to slow down if it is distant from the sun. This behavior is mathematically described in Equation (12), which uses it to calculate an object’s velocity around the sun using the vis viva equation. This equation has two parts as shown below:

(12) $$V_{{}_{i}}(t)=\left\{\begin{array}{c} ({\vec{X}}_{a}-{\vec{X}}_{i})\times r_{4}\times H+F\times {\vec{U}}_{2}\times \left(1-R_{i-norm}(t)\right)\\ \times (r_{3}{\vec{X}}_{i,up}-{\vec{X}}_{i,low}){\vec{r}}_{5}\;if\;R_{i-norm}(t)>0.5\\ (2\times r_{4}\times {\vec{X}}_{i}-{\vec{X}}_{b})\rho +({\vec{X}}_{a}-{\vec{X}}_{b})\rho^{*}\\ +F\times {\vec{U}}_{1}\times \left(1-R_{i-norm}(t)\right)\times ({\vec{X}}_{i,up}-{\vec{X}}_{i,low}){\vec{r}}_{5}\;Else \end{array}\right.$$

where

(13) $$H=\sqrt{(Ms+m_{i})\times \mu (t)\left|-\frac{1}{a_{i}(t)+\varepsilon }+\frac{2}{R_{i}(t)+\varepsilon }\right|}$$

(14) $$\rho =(r_{3}\times (1-r_{4})+r_{4})\times \vec{U}\times H$$

(15) $$\rho^{*}=(r_{3}\times (1-r_{5})+r_{5})\times (1-\vec{U})\times H$$

where *V_i_*(*t*) represents the velocity of object *i* at time *t*; *X_i_* characterizes an object *i*, whilst *F* manifests an integer number chosen randomly that belongs to the set {−1, 1}; the symbol $\vec{U}$ is a vector containing integer number randomly chosen which belongs to the set {0, 1}; *r*_1_, *r*_2_, *r*_3_, *r*_4_, and *r*_5_ denote random integer numbers uniformly distributed in the range [0, 1]; *μ*(*t*) denotes the universal gravitational constant; *X_a_* and *X_b_* signify solutions that are selected from the population at random; *Ms* and *m_i_* characterize the mass of *X_s_* and *X_i_*, respectively; *Ri*(*t*) demonstrates the distance at time *t* among the best solution (*X_s_*) and the object (*X_i_*); *ε* demonstrates a minimal value for avoiding a divide-by-zero mistake; and *a_i_* is the elliptical orbit semimajor axis at time *t* of object *i*, and it is defined by Kepler’s third law as follows:

(16) $$a_{i}(t)={\left[\mu (t)\times (Ms+m_{i})\times \frac{T_{i}{}^{2}}{4\pi^{2}}\right]}^{\frac{1}{3}}\times r_{3}$$

where *T_i_* is an absolute value that is produced at random using the normal distribution to represent the orbital period of object *i*. The semimajor axis of object *i*’s elliptical orbit is considered in our proposed algorithm to steadily decrease over generations as the solutions advance toward the region where the best overall solution is most likely to be discovered. *R_i−norm_*(*t*) denotes normalizing the Euclidian distance among *X_s_* and *X_i_*; its definition is as follows.

(17) $$R_{i-norm}(t)=(R_{i}(t)-R_{\min}(t))/(R_{\max}(t)-R_{\min}(t))$$

### 3.3. Step 3: Escaping from the Local Optimum

Most solar system objects rotate on their own axes and move in an anticlockwise manner around the sun; however, certain objects move in a clockwise motion. This behavior is used by the approach suggested to leave local optimal zones. The suggested KOA simulates this behavior by employing a flag *F* that modifies the search direction such that agents have a good chance of accurately scanning the search space.

### 3.4. Step 4: Updating Objects’ Positions

As previously explained, objects have their own elliptical orbit around the sun. Objects rotate near the sun, becoming closer to it for a while and then moving farther from it. The two main parts of the proposed KOA, which are the exploitation and exploration phases, can simulate this behavior. The KOA searches for new locations near the best solutions while employing solutions close to the sun more precisely and exploring things far from the sun to locate new solutions. The fact that the objects are far from the sun throughout the exploration phase shows that the suggested method efficiently explores the whole search area.

The following equation is used to update the location of each object far from the sun in line with the preceding steps:

(18) $${\vec{X}}_{i}(t+1)={\vec{X}}_{i}(t)+{\vec{V}}_{i}(t)\times F+({\vec{X}}_{s}(t)-{\vec{X}}_{i}(t))\times \vec{U}\times \left(Fg_{i}(t)+\left|r\right|\right)$$

where *X_i_*(*t +* 1) stands for the new location at time *t* + 1 of an object *I*, while *X_i_*(*t*) represents the present location of the object *i* at time *t*; *V_i_*(*t*) illustrates the velocity of object *i* that is needed to transfer to the new location, *X_s_*(*t*) manifests the best sun position which is associated with the best solution that acquires the least fitness score, and *F* is demonstrated as a flag to switch the search’s direction.

The term *Fg_i_* in the context of the Kepler optimization algorithm (KOA) represents the attraction force between the sun (*X_s_*) and any planet (*X_i_*). This force is calculated based on the universal law of gravitation and can be expressed using the following equation:

(19) $$Fg_{i}(t)=r_{4}+\mu (t)\times e_{i}\times ({\vec{Mn}}_{s}\times {\vec{mn}}_{i})/({\vec{Rn}}_{i}{}^{2}+\varepsilon )$$

where *e_i_* manifests the eccentricity of a planet’s orbit, which is a value between 0 and 1 that was proposed to endow a stochastic characteristic to the KOA. Additionally, the normalized values of *m_i_* and *Ms* can be defined by *mn_i_* and *Mn_s_* that demonstrate the mass of *X_i_* and *X_s_*, respectively.

This equation is utilized to model the gravitational force between celestial bodies, specifically the sun and the planets, as part of the algorithm’s calculations. The force is an essential factor in determining the planetary motion and optimizing the trajectories of the planets within the system. By incorporating the sun’s attraction force, the KOA can simulate the gravitational interactions between celestial bodies and effectively optimize the orbits and positions of the planets in the system being studied. This enables the algorithm to provide accurate and efficient solutions for various astronomical and astrophysical problems. The normalized values of *m_i_* and *Ms* can be mathematically represented by Equations (20) and (21):

(20) $$Mn_{s}=r_{2}\times (fit_{s}(t)-worst(t))/\sum_{k=1}^{N_{p}}\left(fit_{k}(t)-worst(t)\right)$$

(21) $$mn_{i}=(fit_{i}(t)-worst(t))/\sum_{k=1}^{N_{p}}\left(fit_{k}(t)-worst(t)\right)$$

where *worst*(*t*) represents the solution candidate with the highest fitness score; *fit_k_*(*t*) indicates the value of the fitness function regarding each location of the object *k* at the current time *t*.

The Euclidian distance between *X_s_* and *X_i_* can be defined by the term (*Rn_i_*), as depicted in Equation (22), which represents the normalized value of (*R_i_*).

(22) $$Rn_{i}(t)={\left\|X_{s}(t)-X_{i}(t)\right\|}_{2}=\sqrt{\left(\sum_{j=1}^{Dim}{\left(X_{s}(t)-X_{i}(t)\right)}^{2}\right)}$$

To manage search accuracy, *μ*(*t*) is defined by Equation (23) as a function that exponentially declines with time (*t*).

(23) $$\mu (t)=\mu_{o}\times e^{-(t/T_{\max})\gamma }$$

where *μ_o_* is an initial value; *γ* is a constant; and *T_max_* and *t* demonstrate the maximum iterations’ number and current iteration number, respectively.

### 3.5. Step 5: Updating Distance with the Sun

The normal distance behavior, which normally fluctuates with time between the planets and the sun, is simulated to further enhance the exploitation and exploration operators of planets. The KOA will concentrate on optimizing the exploitation operator when planets are near the sun and the exploration operator when the sun is far away. This principle is represented mathematically as follows:

(24) $${\vec{X}}_{i}(t+1)={\vec{U}}_{1}\times {\vec{X}}_{i}(t)+\left(1-{\vec{U}}_{1}\right)\times \left({\vec{X}}_{i}(t)+{\vec{X}}_{a}(t)+{\vec{X}}_{s})/3+\frac{1}{e^{\left(r\times \left(1+r_{4}\times (a_{2}-1)\right)\right)}}\times \left(\left({\vec{X}}_{i}(t)+{\vec{X}}_{a}(t)+{\vec{X}}_{s})/3-{\vec{X}}_{b}(t\right)\right)\right)$$

where *a*_2_ defines a cyclic controlling parameter as manifested in Equation (25):

(25) $$a_{2}=-(1+t/T_{\max})$$

### 3.6. Step 6: Elitism

**Figure 2:**
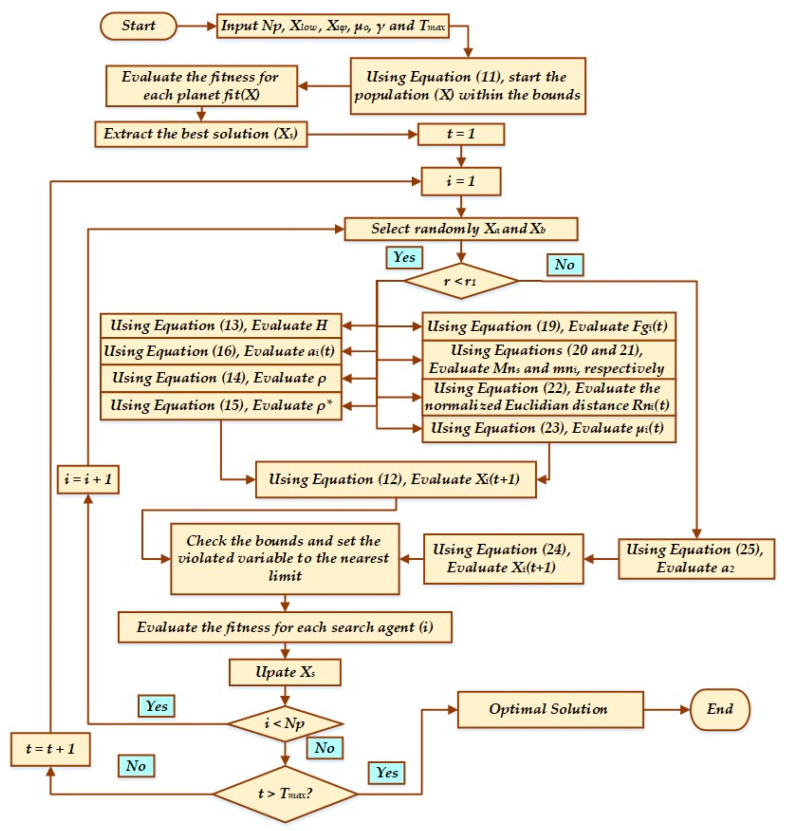
Flowchart of KOA.

This stage employs an elitist method in order to guarantee the optimal placements for the sun and the planets. Equation (26) provides a summary of this process. Figure 2 describes in detail the flowchart of KOA.

(26) $${\vec{X}}_{i,new}(t+1)=\left\{\begin{array}{cc} {\vec{X}}_{i}(t+1) & if\;fit\left({\vec{X}}_{i}(t)\right)\geq fit\left({\vec{X}}_{i}(t+1)\right)\\ {\vec{X}}_{i}(t) & Else \end{array}\right.$$

## 4. Performance of KOA on the CHPUED Issue

In this section, the proposed KOA is tested on large 48-unit, 96-unit, and 192-unit test systems to demonstrate its efficacy and superiority when handing the CHPED issue.

### 4.1. The 48-Unit System

The system, in this instance, involves 48 total units, including 10 power–heat combination units, 12 power-only units, and 26 heat-only units. It is necessary, in this instance, to provide 2500 MWth of heat and 4700 MW of power. Additionally, the valve-point impact for power-only units is taken into account. The capacity limits of heat-only and power-only units, as well as the cost coefficients of associated units, are taken from Reference [19]. Table 1 presents the test outcomes of all units obtained using the KOA.

The output power of the power-only units (MW) is reflected by parameters between P1 and P104. P105 and P152 are the power outputs of CHP units in MW and H105 and H152 relate to heat outputs of CHP units in MWth. Additionally, H153 and H192 are the outputs of heat-only units in MWth. As can be seen in Table 1, the Sum (Hg) and Sum (Pg) values satisfy the heat and power demands of 2500 MWth and 4700 MW, respectively. As demonstrated from Table 1, the best cost value is identified by the KOA as 116,650.0870. Additionally, all results are in the feasible zone, and several individual findings are put exactly at the lower or higher bounds.

**Figure 3:**
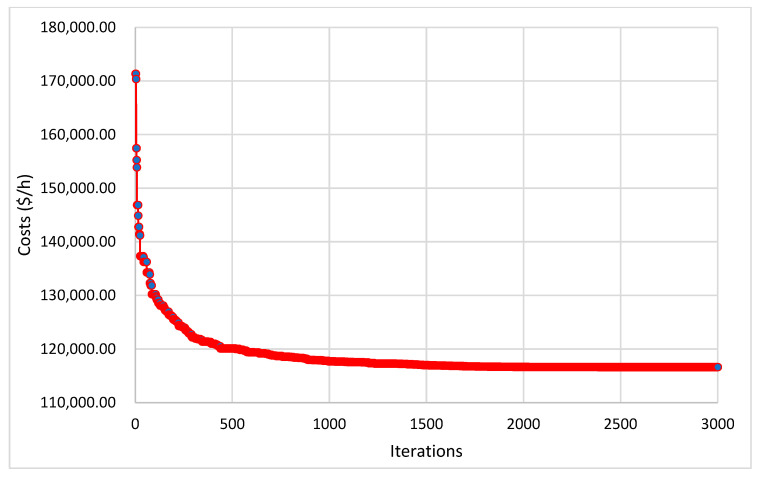
Convergence rates of the proposed KOA for 48-unit CHPUED system.

Figure 3 illustrates the suggested KOA’s convergence rates for the given system, where the curve of the proposed KOA converges quickly. The proposed KOA requires around 2400 iterations to obtain the best solution. The results show that the proposed KOA has outstanding convergence rates for the given large CHPUED system.

Table 2 presents a comprehensive comparison between the proposed KOA and various other techniques reported in the literature. The comparison is conducted on a 48-unit CHPUED test system. The techniques included in the comparison are supply–demand optimization (SDO) [27], the multi-verse algorithm (MVA) [28], the gravitational search algorithm (GSA) [29], civilized swarm optimization (CSO) incorporated Powell’s pattern search (PPS) [30], gray wolf optimization (GWO) [28], the salp swarm algorithm (SSA) [31], the GSO-based algorithm with ranger operators and modified scrounger (MGSO) [32], CPSO [19], differential evolution (DE) [28], the crow search algorithm (CSA) [28], the marine predator algorithm (MPA) [33], the jellyfish search optimizer (JFSO) [34], the manta ray foraging algorithm (MRFA) [28], and PSO with time-varying acceleration coefficients (PSO-TVAC) [19].

Table 2 clearly demonstrates that the proposed KOA outperforms all the other optimizers in terms of performance and cost. It attains the most favorable results among the compared techniques, making it the superior choice for CHPUED optimization. The KOA exhibits the lowest minimum, standard deviation, average, and worst values of 116,650.0870, 298.8796, 117,104.5447, and 117,915.5359, respectively, as indicated in Table 2. This table clearly shows that the proposed KOA has the best performance and the lowest cost of these optimizers. Moreover, this comparison validates the proposed KOA’s efficacy and superiority when used with CHPUED. Furthermore, the proposed KOA also receives the lowest minimum, standard deviation, average, and worst values of 116,650.0870, 298.8796, 117,104.5447, and 117,915.5359 $, as shown in Table 2. Consequently, the proposed KOA has greater robustness than the techniques that have been reported. Based on the best attained costs, the proposed KOA shows improvements of 3.660%, 1.982%, 0.589%, 0.864%, 3.021%, 0.180%, 2.680%, 5.404%, 0.614%, 3.286%, 5.086%, 0.615% and 0.613%, respectively, compared to the following algorithms and optimization methods: CPSO [19], PSO-TVAC [19], MRFA [28], MVA [28], SSA [31], MPA [33], GSA [29], CSA [28], MGSO [32] DE [28], GWO [28], CSO, PPS [30] and JFSO [34]. These statistics further reinforce the robustness of the proposed KOA compared to the reported techniques.

The comprehensive analysis presented in Table 2 serves to validate the efficacy and superiority of the proposed KOA specifically when applied to the CHPUED problem. Its exceptional performance, combined with the lowest cost values, establishes the KOA as the most reliable and effective optimization approach within the context of CHPUED.

### 4.2. The 96-Unit System

The system, in this instance, involves 96 total units, including 24 power–heat combination units, 52 power-only units, and 20 heat-only units. It is necessary, in this instance, to provide 5000 MWth of heat and 9400 MW of power. Additionally, the valve-point impact for power-only units is taken into account. The capacity limits of heat-only and power-only units, as well as the cost coefficients of associated units, are taken from Reference [24]. Table 3 presents the test outcomes of all units obtained using the KOA.

H1 and H24 relate to heat outputs of CHP units in MWth, P53 and P76 are the power outputs of CHP units in MW, and between H25 and H44 are the outputs of heat-only units in MWth. The output power of the power-only units (MW) is reflected by the parameters between P1 and P52. Additionally, Sum (Hg), Sum (Pg), and WCTF stand for the total heat production (MWth), total power generation (MW), and total generation costs ($) of thermal electrical and energy, respectively. As can be seen in Table 3, the Sum (Hg) and Sum (Pg) values satisfy the heat and power demands of 5000 MWth and 9400 MW, respectively. The best cost value is identified by KOA as 234,285.3. Additionally, all results are in the feasible zone, and several individual findings are put exactly at the lower or higher bounds. Additionally, the standard HBA, the standard JSA, and the proposed HBJSA effectively achieve all constraints with 100% accuracy, as illustrated in Table 3.

**Figure 4:**
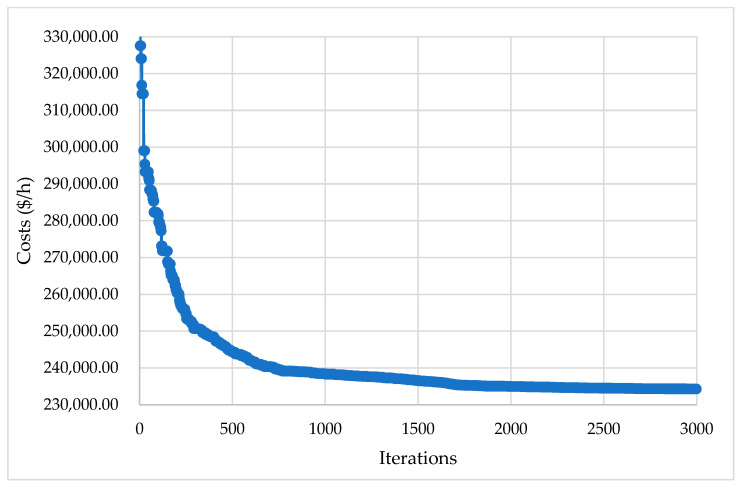
Convergence rates of the proposed KOA for 96-unit CHPUED system.

Figure 4 illustrates the suggested KOA’s convergence rates for the given system, where the curve of the proposed KOA converges quickly. The proposed KOA requires around 2700 iterations to obtain the best solution. The results show that the proposed KOA has outstanding convergence rates for the given large CHPUED system.

A comparison between the proposed KOA and other reported techniques is depicted in Table 4. The reported techniques that are employed for the 96-unit CHPUED test system are the whale optimization algorithm (WOA) [24], supply–demand optimization (SDO) [27], the marine predator algorithm (MPA) [33], the improved MPA (IMPA) [33], the heap technique (HT) [34], the jellyfish search optimizer (JFSO) [34], hybrid HTJFSO (HHTJFSO) [34], the manta ray foraging algorithm (MRFA) [28], weighted vertices optimization and PSO (WVO-PSO) [35], and PSO with time-varying acceleration coefficients (PSO-TVAC) [19]. This table clearly shows that the proposed KOA has the best performance and the lowest cost of these optimizers. Moreover, this comparison validates the proposed KOA’s efficacy and superiority when used with CHPUED. Furthermore, the proposed KOA also receives the lowest minimum, standard deviation, average, and worst values of 234,285.2584, 761.7006, 235,683.2917, and 236,929.2188. Consequently, the proposed KOA has greater robustness than the techniques that have been reported. Based on the best attained costs, the proposed KOA derives improvements of 0.423%, 0.349%, 0.235%, 1.030%, 0.416%, 0.853%, 2.072%, 1.588%, 2.807% and 0.811%, respectively, compared to the following algorithms and optimization techniques: JFSO [34], HT [34], HHTJFSO [34], WOA [24], IMPA [33], MPA [33], PSO-TVAC [19], WVO-PSO [35], WVO [35] and SDO [27]. These statistics further reinforce the robustness of the proposed KOA compared to the reported techniques.

The extensive examination depicted in Table 4 provides substantial evidence to support the effectiveness and superiority of the proposed Kepler optimization algorithm (KOA) in addressing the CHPUED problem. The remarkable performance of the KOA, coupled with its cost-effectiveness, solidifies its position as the most dependable and efficient optimization approach for CHPUED applications.

### 4.3. The 192-Unit System

The system, in this instance, involves 192 total units, including 48 power–heat combination units, 104 power-only units, and 40 heat-only units. It is necessary, in this instance, to provide 10,000 MWth of heat and 18,800 MW of power. Additionally, the valve-point impact for power-only units is taken into account. The capacity limits of heat-only and power-only units, as well as the cost coefficients of associated units, are taken from Reference [24].

Table 5 presents the obtained objective costs of the proposed KOA, the dwarf mongoose optimization algorithm (DMOA) [36], the energy valley optimizer (EVO) [37], GWO and PSO. The best cost value is identified by the KOA as 487,145.2. As demonstrated from Table 5, KOA acquires a value of 487,145.2, whereas the DMOA [36], the EVO [37], GWO, and PSO have values of 581,798, 572,324.8, 678,051.9, 793,224.8, respectively. According to the obtained costs, the presented KOA successfully achieves improvement of 19.43%, 17.49%, 39.19% and 62.83% compared to the DMOA, the EVO, GWO and PSO, respectively.

Also, the detailed test outcomes of all units obtained using the applied algorithms are tabulated in Appendix A. The output power of the power-only units (MW) is reflected by parameters between P1 and P104. P105 and P152 are the power outputs of CHP units in MW and H105 and H152 relate to heat outputs of CHP units in MWth. Additionally, H153 and H192 are the outputs of heat-only units in MWth. As can be seen in Table 5, the Sum (Hg) and Sum (Pg) values satisfy the heat and power demands of 10,000 MWth and 18,800 MW, respectively.

**Figure 5:**
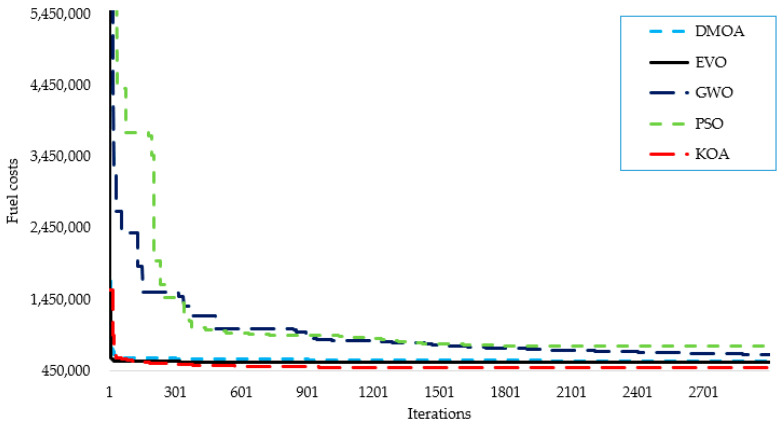
Convergence rates of the proposed KOA, DMOA, EVO, GWO, and PSO for the 192-unit CHPUED system.

**Figure 6:**
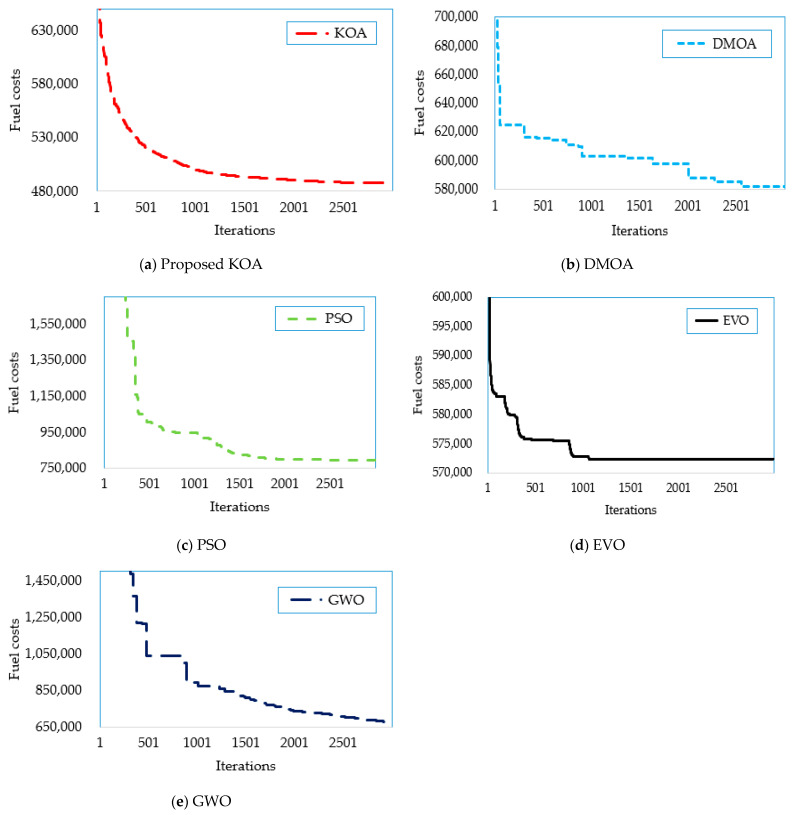
Convergence rates of each individual applied algorithm for the 192-unit CHPUED system.

Figure 5 illustrates the convergence rates of the KOA, the DMOA, the EVO, GWO, and PSO for the given system, where the curve of the proposed KOA converges quickly. Regarding Figure 5, Table 6 presents the detailed setting of parameters for the applied algorithms. As shown in Figure 5, the proposed KOA requires around 1500 iterations to obtain the best solution. The results show that the proposed KOA has outstanding convergence rates over the DMOA, the EVO, GWO, and PSO for the given large CHPUED system. It can be seen from Figure 5 that the DMOA and EVO seem to have a line convergence characteristic from the first to the last iteration. To clarify this point, a close zooming vision is displayed for the convergence rates of each individual applied algorithm in Figure 6. As shown in Figure 6b, the DMOA does not show a line characteristic, but on the contrary, it shows a gradual decrease in the objective. Compared to the KOA in Figure 6a, the proposed KOA shows a smooth converging feature, while the DMOA convergence is unsmooth like stairs. On the other side, the EVO starts searching and finding solutions to minimize the objective. Unfortunately, it derives a straight line after approximately half of the iterations’ journey. This indicates that this method is stuck in a local minimum. This analysis illustrates the significant convergence characteristics of the proposed KOA against the DMOA, the EVO, GWO, and PSO.

To display the simulation time, Table 7 records the average time per each iteration of the proposed KOA for all cases. As shown in this table, the simulation time for the large-scale system of 192 units is 0.1399 s, where it records 0.0952 for the 48-unit system with time increased by more than 32%. The more the scalability of the case study under investigation increases, the more simulation time is required. By utilizing the well-known Big O notation, the computational complexity of the applied algorithm can be estimated by multiplying the number of design variables, number of solutions and maximum number of iterations. Based on that, the computational complexity for each case study is recorded in Table 8.

### 4.4. Feasibility Study for 192-Unit System

**Figure 7:**
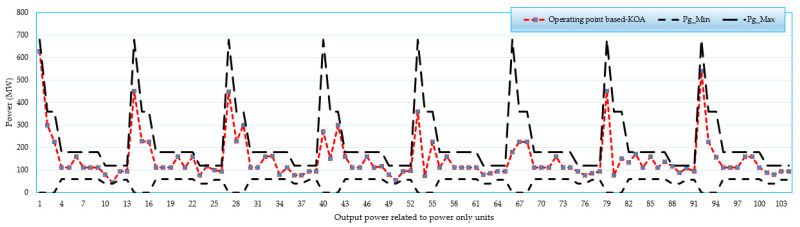
Operating points related to power-only units.

**Figure 8:**
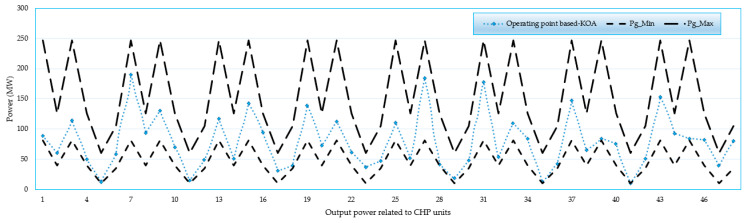
Operating points related to CHP units in terms of their output power.

**Figure 9:**
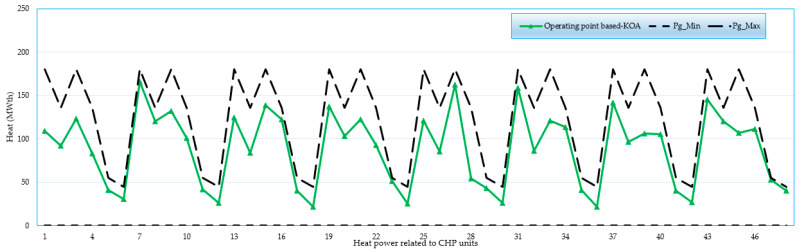
Operating points related to CHP units in terms of their output heat.

**Figure 10:**
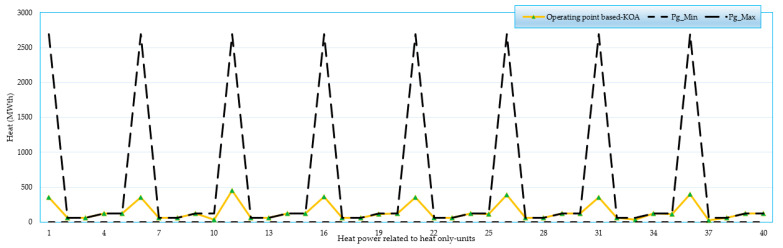
Operating points related to heat-only units.

A feasibility study is conducted for the 192-unit test system when applying the KOA. Figure 7, Figure 8, Figure 9 and Figure 10 display the operating points related to power-only units, CHP units and heat-only units, respectively. As illustrated, all operating points are found between the boundaries of power-only, heat-only, and CHP units. These results demonstrate the effectiveness of the proposed KOA in obtaining practical feasible solutions without any violations. All results are in the feasible zone, and several individual findings are put exactly at the lower or higher bounds. Furthermore, as shown in Figure 7, Figure 8, Figure 9 and Figure 10, the proposed KOA completely and accurately satisfies all criteria.

### 4.5. Discussion

In the previous subsections, the proposed KOA is tested on large 48-unit, 96-unit, and 192-unit test systems. Different remarks are discussed, which are summarized in the following paragraphs.

Table 1 and Table 3 and Appendix A provide a comprehensive depiction of the operating points of the power-only, CHP, and heat-only units for all units in the three investigated systems. These tables effectively describe and demonstrate that the operating points of all units, which are categorized as power-only, CHP, and heat-only, are maintained within the specified limits. This serves as evidence that the proposed KOA successfully preserves the operating points within the defined boundaries.

The convergence rates of the suggested KOA are visualized in Figure 3, Figure 4 and Figure 6 for the three investigated systems. These figures vividly illustrate the high-quality and rapid response of the KOA’s convergence rates. The plots demonstrate the algorithm’s ability to quickly converge toward optimal solutions, indicating its efficiency and effectiveness.

Table 2, Table 4 and Table 5 present various comparisons between the proposed KOA and other reported techniques for the three investigated systems. The extensive analysis provided in these tables showcases the exceptional performance and cost-effectiveness of the proposed KOA when compared to alternative methods. The KOA not only achieves superior results in terms of CHPUED optimization, but it also exhibits greater robustness compared to its counterparts. These findings strongly validate the credibility and value of the proposed KOA as an efficient and reliable optimization solution for CHPUED applications.

For the large 192-unit test system, a feasibility study is conducted and analyzed using Figure 7, Figure 8, Figure 9 and Figure 10. These figures demonstrate that all operating points fall within the boundaries of power-only, heat-only, and CHP units. The results illustrate the effectiveness of the proposed KOA in obtaining practical and feasible solutions while avoiding any violations. All the obtained results lie within the feasible zone, and several individual findings precisely align with the lower or upper bounds. Furthermore, as depicted in Figure 7, Figure 8, Figure 9 and Figure 10, the proposed KOA satisfactorily fulfills all the defined criteria, demonstrating its accuracy and compliance with the given constraints.

## 5. Conclusions

### 5.1. Paper’s Findings

In this study, the KOA is developed for the non-convex CHPUED issue. Kepler’s laws of planetary motion serve as the main source of inspiration for the KOA. According to Kepler’s laws, four operators—position, gravitational force, mass, and orbital velocity—affect how planets move around the sun. To demonstrate the effectiveness of the proposed KOA methodology, three test CHPUED systems are chosen, which are 48, 96, and 192-unit systems. Additionally, new optimizers are introduced for the large 192-unit test system, which are the DMOA, the EVO, GWO, and PSO. A feasibility study is conducted, which demonstrates the superiority and robustness of the proposed KOA. Furthermore, the proposed KOA delivers the lowest overall cost values for the three test systems when compared with various well-known methodologies that have been presented in the scientific literature and the new approaches that are implemented for the first time in this research.

### 5.2. Future Works

The proposed Kepler optimization algorithm (KOA) demonstrates significant potential for effectively addressing the CHPUED problem in large-scale systems. There is room for further enhancement in the system’s performance.

- One potential area of enhancement is to upgrade the model by incorporating external market signals.
- Integrating external factors and signals from the market can help determine the optimal dispatch scenario.
- The constraints of the transmission losses can be considered, which add more complexity to the study.
- The scope of the work can be expanded by incorporating the emission dispatch of thermal units.
- Considering the environmental impact and emissions of the thermal units can lead to more sustainable and environmentally friendly dispatch solutions.
- The integration of renewable energies should be considered for future extensions of the work, promoting a greener and more sustainable energy mix.

## Figures and Tables

**Table 1:** Test outcomes of all units for the 48-unit test system obtained using the KOA.

Unit	KOA	Unit	KOA	Unit	KOA
Pg1	448.812	Pg22	109.8988	Hg31	40.81127
Pg2	299.5241	Pg23	77.46306	Hg32	26.16548
Pg3	150.1696	Pg24	40.16544	Hg33	111.9561
Pg4	159.738	Pg25	92.93015	Hg34	91.23516
Pg5	109.9199	Pg26	55.37272	Hg35	115.2107
Pg6	159.8738	Pg27	91.53579	Hg36	101.8837
Pg7	110.3709	Pg28	45.4442	Hg37	40.00348
Pg8	159.7404	Pg29	91.34668	Hg38	26.99712
Pg9	109.939	Pg30	53.92644	Hg39	418.7711
Pg10	77.48522	Pg31	11.89511	Hg40	59.99864
Pg11	77.51255	Pg32	48.56369	Hg41	59.99945
Pg12	94.00181	Pg33	93.75923	Hg42	119.9865
Pg13	92.45571	Pg34	58.81029	Hg43	119.9993
Pg14	448.9186	Pg35	99.55169	Hg44	418.9729
Pg15	224.4459	Pg36	71.14285	Hg45	59.99973
Pg16	225.5116	Pg37	10.01567	Hg46	59.99715
Pg17	109.8786	Pg38	50.40061	Hg47	119.9984
Pg18	109.877	Hg27	110.7085	Hg48	119.9969
Pg19	109.9607	Hg28	79.69631	Sum (Pg)	4700
Pg20	159.7711	Hg29	110.5908	Sum (Hg)	2500
Pg21	159.871	Hg30	87.02126	WCTF ($)	116,650.0870

**Table 2:** Comparison of the proposed KOA with the reported techniques for 48-unit system.

Optimizer	Min (WCTF ($))	Improving Percentages	Average	Average	Worst	Standard Deviation (Std)
KOA	116,650.0870	-	117,104.5447	117,104.5447	117,915.5359	298.8796
CPSO [19]	120,918.9	3.660%	-	-	-	-
PSO-TVAC [19]	118,962.5	1.982%	-	-	-	-
MRFA [28]	117,336.9	0.589%	-	-	-	-
MVA [28]	117,657.9	0.864%	-	-	-	-
SSA [31]	120,174.1	3.021%	-	-	-	-
MPA[33]	116,860.6	0.180%	-	-	-	-
GSA [29]	119,775.9	2.680%	-	-	-	-
CSA [28]	122,953.5	5.404%	-	-	-	-
MGSO [32]	117,366.09	0.614%	-	-	-	-
DE [28]	120,482.7	3.286%	-	-	-	-
GWO [28]	122,583.3	5.086%	-	-	-	-
CSO and PPS [30]	117,367.09	0.615%	-	-	-	-
JFSO [34]	117,365.09	0.613%	-	-	-	-

**Table 3:** Test outcomes of all units for the 96-unit test system obtained using the KOA.

Unit	KOA	Unit	KOA	Unit	KOA	Unit	KOA
Pg1	538.7944	Pg32	110.0436	Pg63	10.02797	Hg70	21.0867
Pg2	299.6272	Pg33	110.3995	Pg64	48.32375	Hg71	109.8662
Pg3	151.2914	Pg34	110.1027	Pg65	82.02374	Hg72	88.07219
Pg4	109.8576	Pg35	159.8276	Pg66	44.18677	Hg73	123.5494
Pg5	109.9343	Pg36	78.60966	Pg67	85.76875	Hg74	99.22525
Pg6	109.8765	Pg37	77.69705	Pg68	73.52597	Hg75	41.11163
Pg7	160.1471	Pg38	57.87178	Pg69	10.29498	Hg76	28.39532
Pg8	110.2941	Pg39	94.20503	Pg70	37.45466	Hg77	408.4901
Pg9	110.342	Pg40	359.9866	Pg71	90.09144	Hg78	59.94804
Pg10	81.05786	Pg41	149.6621	Pg72	55.19146	Hg79	59.99218
Pg11	78.13509	Pg42	149.9737	Pg73	114.497	Hg80	119.9246
Pg12	92.73616	Pg43	110.1571	Pg74	68.07549	Hg81	119.9496
Pg13	92.42638	Pg44	110.1453	Pg75	12.65002	Hg82	410.5949
Pg14	628.4539	Pg45	159.932	Pg76	53.60396	Hg83	59.97848
Pg15	224.6278	Pg46	111.2773	Hg53	124.1457	Hg84	59.97649
Pg16	224.6525	Pg47	160.9166	Hg54	81.36933	Hg85	119.9758
Pg17	109.9961	Pg48	110.255	Hg55	132.9305	Hg86	119.9933
Pg18	159.7635	Pg49	79.55363	Hg56	79.41519	Hg87	405.6305
Pg19	161.739	Pg50	77.98886	Hg57	41.42439	Hg88	58.02127
Pg20	159.8074	Pg51	92.93884	Hg58	31.21828	Hg89	59.99075
Pg21	110.7902	Pg52	92.42203	Hg59	109.8515	Hg90	119.9301
Pg22	110.1921	Pg53	115.5219	Hg60	88.44604	Hg91	119.9778
Pg23	40.25017	Pg54	47.40113	Hg61	131.9541	Hg92	411.7448
Pg24	77.45754	Pg55	131.1481	Hg62	92.84449	Hg93	59.9785
Pg25	93.03079	Pg56	45.14028	Hg63	39.99935	Hg94	59.98367
Pg26	93.02481	Pg57	13.32731	Hg64	26.01246	Hg95	119.9128
Pg27	269.3372	Pg58	59.77202	Hg65	105.3056	Hg96	119.8671
Pg28	224.586	Pg59	90.13865	Hg66	78.57885	Sum (Pg)	9400
Pg29	299.8866	Pg60	55.61345	Hg67	107.3423	Sum (Hg)	5000
Pg30	109.851	Pg61	129.425	Hg68	103.879	WCTF ($)	234,285.3
Pg31	160.1618	Pg62	60.70201	Hg69	40.11558		

**Table 4:** Comparison of the proposed KOA with the reported techniques for 96-unit system.

Optimizer	(WCTF ($/h))	Improving Percentages	Average	Worst	Std
KOA	234,285.2584	-	235,683.2917	236,929.2188	761.7006
JFSO [34]	235,277.05	0.423%	236,688.7625	237,940.189	859.1088
HT [34]	235,102.65	0.349%	236,853.3030	239,119.459	1594.7970
HHTJFSO [34]	234,836.04	0.235%	235,646.1289	236,967.064	764.9310
WOA [24]	236,699.15	1.030%	237,431.4678	238,877.049	971.5473
IMPA [33]	235,260.3	0.416%	-	-	-
MPA [33]	236,283.1	0.853%	-	-	-
PSO-TVAC [19]	239,139.5018	2.072%	-	-	-
WVO-PSO [35]	238,005.79	1.588%	-	-	-
WVO [35]	240,861.3210	2.807%	-	-	-
SDO [27]	236,185.18	0.811%	-	-	-

**Table 5:** Obtained costs of the KOA, DMOA, EVO, GWO, and PSO for the large 192-unit system.

Algorithm	DMOA	EVO	GWO	PSO	KOA
WCTF ($)	581,798	572,324.8	678,051.9	793,224.8	487,145.2
Improving Percentages	19.43%	17.49%	39.19%	62.83%	-

**Table 6:** Setting of parameters for the applied algorithms for the large 192-unit system.

Algorithm	Parameter Settings
KOA	*μ_o_* = 0.1; *γ* = 15; number of solutions = 100; maximum number of iterations = 3000.
DMOA	number of babysitters = 3; number of alpha group = 97; number of scouts = 97; babysitter exchange parameter = 431; alpha female—vocalization = 2; number of solutions = 100; maximum number of iterations = 3000.
EVO	number of solutions = 100; maximum number of iterations = 3000.
GWO	number of solutions = 100; maximum number of iterations = 3000.
PSO	cognitive parameter (*c*1) = 2; social parameter (*c*2) = 2; number of solutions = 100; maximum number of iterations = 3000.

**Table 7:** Simulation time of the proposed KOA for all cases.

Case Study	Average Time Per Iteration (Seconds)
48-unit CHPUED system	0.0952
96-unit CHPUED system	0.0981
192-unit CHPUED system	0.1399

**Table 8:** Computational complexity of the applied algorithm.

Test Case	Dimension	Number of Solutions	Maximum Number of Iterations	Computational Complexity
48-unit test system	60	100	3000	O (1,800,000)
96-unit test system	120	100	3000	O (3,600,000)
192-unit test system	240	100	3000	O (7,200,000)

## Data Availability

Data are contained within the article.

## Appendix A

Table A1 and Table A2 record the detailed test outcomes of all units obtained using the applied algorithms for the 192-unit system. Table A1 displays the output electrical powers from power-only units regarding the KOA, the DMOA, the EVO, GWO, and PSO. Also, Table A2 displays the output electrical and heat powers from CHP and heat-only units regarding the KOA, the DMOA, the EVO, GWO, and PSO.

**Table A1:** Output electrical powers from power-only units regarding KOA, DMOA, EVO, GWO, and PSO for the 192-unit system.

Unit	DMOA	EVO	GWO	PSO	KOA	Unit	DMOA	EVO	GWO	PSO	KOA
Pg1	174.4182	448.799	628.4634	539.1442	629.1331	Pg61	62.98548	127.446	160.1939	60	110.3749
Pg2	244.4594	319.5189	8.117312	360	300.1081	Pg62	94.045637	73.04489	48.89186	40	78.26122
Pg3	246.9179	224.7471	85.90084	359.9992	224.2466	Pg63	95.095479	82.58672	76.54153	40	84.58004
Pg4	163.9458	70.75565	131.4979	60	110.3729	Pg64	116.50456	58.24736	64.01384	55	92.5379
Pg5	127.0533	114.4128	174.8349	179.9989	109.9616	Pg65	112.32882	92.2212	115.9772	120	94.09216
Pg6	97.80373	110.3963	109.8681	60	159.7144	Pg66	386.54269	628.3049	635.5608	1.6574276	180.5931
Pg7	82.64369	136.3851	151.7705	60	110.5713	Pg67	219.58848	123.3935	4.23799	360	224.9961
Pg8	122.0835	90.59523	62.34392	180	109.7013	Pg68	178.59082	175.2344	310.7114	0	225.5173
Pg9	165.1865	104.3483	159.8432	180	110.1922	Pg69	179.71952	126.2191	126.4011	100.46434	110.8168
Pg10	89.35626	85.93036	70.06314	40	78.27927	Pg70	100.59707	137.768	78.02074	180	110.6743
Pg11	84.92388	85.12978	96.43349	120	43.99838	Pg71	81.052792	137.9082	155.4551	60	110.4331
Pg12	98.99822	88.60448	118.4218	55	92.8743	Pg72	120.96628	113.491	65.80614	180	160.4197
Pg13	94.462	96.68395	85.69435	55	92.79832	Pg73	139.2029	133.8202	160.5635	180	110.4285
Pg14	425.6213	263.8784	628.0853	680	450.1082	Pg74	127.74529	122.1182	175.8073	60.036551	111.1456
Pg15	233.561	149.756	330.4895	0	227.5378	Pg75	58.032026	89.98149	79.68891	120	93.31288
Pg16	168.8089	153.1829	359.9657	0	224.0146	Pg76	50.446032	70.14322	88.16762	76.721665	77.00711
Pg17	132.6032	91.9275	159.9322	180	110.0895	Pg77	75.188085	95.22196	75.94925	55	84.51632
Pg18	90.41802	111.1778	167.999	65.02851	109.5931	Pg78	75.090964	66.16529	119.7468	120	92.51662
Pg19	152.9372	103.9256	170.1467	60	110.5872	Pg79	396.90259	448.9468	628.5149	679.99984	451.8399
Pg20	113.8235	135.956	108.5677	60	160.5694	Pg80	54.831829	156.3718	299.5566	0	76.45995
Pg21	102.4749	152.448	61.09929	180	111.0999	Pg81	192.45143	141.0076	357.2925	0	150.0747
Pg22	116.5048	136.4479	107.4706	180	160.08	Pg82	137.34809	111.0114	139.106	60	132.3369
Pg23	98.57747	77.61179	95.16397	120	76.57349	Pg83	102.39792	124.031	92.66801	160.82869	170.2779
Pg24	69.99943	69.24365	69.49135	120	115.0327	Pg84	124.88114	149.3372	72.11485	180	110.7002
Pg25	74.98295	58.11324	118.7751	55	97.8527	Pg85	140.97606	113.9025	113.3606	179.97632	160.294
Pg26	107.4874	93.90026	62.83023	120	93.89541	Pg86	98.353789	158.0664	157.7157	60	111.7676
Pg27	130.2818	393.9314	628.8686	0	448.5537	Pg87	82.70602	104.2559	61.37461	60	136.1275
Pg28	202.8742	251.2048	4.105242	360	227.8284	Pg88	70.062991	99.80671	74.94761	120	115.7378
Pg29	101.974	224.3351	309.367	0	299.847	Pg89	101.70538	91.36633	45.4811	120	88.38295
Pg30	149.0897	107.5555	161.4966	60	110.2662	Pg90	96.584487	59.95746	63.64475	120	108.3084
Pg31	124.6886	138.9449	160.8727	180	111.8424	Pg91	92.112756	91.97635	94.04333	55	92.86743
Pg32	93.87523	110.1144	95.53187	180	160.1724	Pg92	646.61874	110.7548	678.4753	680	539.4136
Pg33	78.43197	151.2344	70.05601	180	162.0457	Pg93	256.19755	223.3503	302.8407	357.30215	225.2475
Pg34	154.356	109.3927	68.09066	167.6068	80.54356	Pg94	220.09345	184.7615	1.312341	0	157.8272
Pg35	86.91799	114.6454	107.543	180	110.7539	Pg95	113.48862	106.7757	126.5713	180	110.1955
Pg36	102.368	63.95323	42.15703	40	77.67327	Pg96	93.56559	159.4062	155.0532	180	110.6043
Pg37	85.88163	74.68514	45.2863	40.3999	77.37991	Pg97	153.0745	133.3575	142.1973	65.613323	110.3772
Pg38	90.14782	91.10007	73.3533	57.69111	92.37051	Pg98	91.853495	97.28445	71.5857	61.951258	159.7578
Pg39	106.8801	96.7277	83.8524	120	92.36988	Pg99	105.02995	130.9669	161.0273	180	159.6356
Pg40	537.0964	353.6418	0	0	269.7407	Pg100	107.11788	124.7339	159.7256	180	109.8936
Pg41	226.4437	317.6857	0	7.252238	149.7111	Pg101	87.902036	83.01983	88.0214	120	87.91491
Pg42	196.0879	148.9983	1.939074	359.9946	299.1913	Pg102	72.763196	87.04375	53.99422	120	78.05281
Pg43	136.7817	141.4821	66.73899	60.12266	159.7018	Pg103	88.142268	87.24567	119.919	55	92.51872
Pg44	62.4416	137.3353	146.7645	60.02225	109.5677	Pg104	101.25252	99.1737	62.69223	120	93.32982
Pg45	80.90206	93.01569	171.1177	180	110.3502	Pg105	184.28464	150.6826	210.538	136.23557	88.70108
Pg46	130.3269	139.3121	109.8648	60	159.8408	Pg106	61.687297	72.20073	103.9249	125.8	59.9183
Pg47	148.1692	109.4169	135.5804	60	109.9596	Pg107	139.87923	132.0641	100.0922	129.94934	114.4677
Pg48	108.8255	131.6218	158.033	60	115.596	Pg108	101.89348	63.19561	79.98875	60.779838	49.69122
Pg49	60.06298	43.3716	53.12575	40	80.30361	Pg109	15.867864	31.36009	29.45192	33.066112	12.32701
Pg50	50.61262	77.5151	70.16409	120	52.28408	Pg110	66.056337	79.13215	37.64521	81.821153	58.4113
Pg51	79.94245	83.08679	60.41968	120	93.1087	Pg111	147.16246	138.5883	111.4306	247	190.3511
Pg52	97.30817	68.1	75.10253	87.41094	95.29422	Pg112	74.170207	92.37211	44.82001	44.277967	93.70398
Pg53	352.3213	448.4259	628.8927	679.9969	359.0342	Pg113	134.13176	165.2974	212.0918	147.7214	130.6392
Pg54	160.1921	150.6065	24.29994	0	74.73825	Pg114	78.339142	76.64612	47.54094	125.8	70.07984
Pg55	338.1157	155.9095	0.016443	0	225.1658	Pg115	34.486175	28.88131	34.25237	60	14.32984
Pg56	117.1937	128.2089	178.8473	60	109.797	Pg116	81.471568	89.49547	54.88681	105	49.16335
Pg57	104.443	162.2053	60.65029	180	159.9216	Pg117	178.77234	156.8938	205.7086	211.52341	116.8013
Pg58	83.74165	158.3383	162.6725	180	109.8658	Pg118	68.201565	78.05904	46.15124	125.8	50.50524
Pg59	107.3655	110.4594	72.58097	60	110.0816	Pg119	182.59667	178.6734	116.2796	208.65061	142.3482
Pg60	127.2445	109.6603	142.5323	60	109.4778	Pg120	96.392192	88.2642	54.64609	125.8	94.62255

**Table A2:** Output electrical and heat powers from CHP and heat-only units regarding KOA, DMOA, EVO, GWO, and PSO for the 192-unit system.

Unit	DMOA	EVO	GWO	PSO	KOA	Unit	DMOA	EVO	GWO	PSO	KOA
Pg121	46.02383	29.3764	28.42926	20.77384	30.97887	Hg134	19.85664	14.6324	6.080416	0	25.88826
Pg122	81.91063	78.77847	37.69174	104.7777	39.40866	Hg135	87.6911	118.0721	0.286583	0	158.9446
Pg123	154.5708	183.7391	185.5751	104.2847	139.0125	Hg136	2.362165	70.79715	5.456858	0	86.28956
Pg124	58.05261	58.466	55.06693	125.8	72.83244	Hg137	116.8887	57.96175	0.036243	0	120.7795
Pg125	96.61376	132.3242	105.6578	101.5627	112.6719	Hg138	99.47672	89.91044	0.419529	92.00018	113.2803
Pg126	100.9668	90.37526	45.05289	48.94307	60.97844	Hg139	22.01637	7.048723	33.90125	44.64996	40.9007
Pg127	24.82604	34.28892	27.31897	20.4968	36.45769	Hg140	17.93339	24.31242	22.88953	0	21.52482
Pg128	89.81316	66.96374	38.96679	58.13628	47.02069	Hg141	110.6448	75.9682	0.004297	135.4516	142.0406
Pg129	158.1581	177.8827	193.223	131.0643	110.6598	Hg142	4.608817	42.24832	95.49187	0	96.56051
Pg130	57.72706	57.7503	53.81889	125.8	52.14506	Hg143	43.61712	65.07744	0.002121	0	106.3423
Pg131	180.7866	134.9287	110.2631	167.5046	184.2545	Hg144	80.59267	86.44938	81.43766	0	105.7199
Pg132	97.75464	72.69634	62.23115	92.44643	41.51712	Hg145	31.65104	23.16603	52.01338	0	39.85337
Pg133	16.4573	39.7243	27.21387	20.30628	18.17227	Hg146	11.1955	21.857	0.014052	0	27.14903
Pg134	51.8256	59.58369	43.36524	105	48.08117	Hg147	124.0557	80.31409	1.030762	133.9085	145.2778
Pg135	133.7342	126.1696	104.0743	247	177.8935	Hg148	53.37894	77.98092	132.4316	0	120.2272
Pg136	53.75736	70.05483	66.04534	62.12208	53.19527	Hg149	111.1171	69.60678	0.080402	0	106.5817
Pg137	186.328	160.1726	106.0133	247	109.8631	Hg150	76.90896	76.83802	61.43779	77.63081	111.0589
Pg138	81.15719	57.42499	54.55075	60.01059	84.42479	Hg151	43.16865	32.22891	12.89287	44.51353	52.44464
Pg139	33.65432	40.93528	20.1056	21.06007	12.26503	Hg152	29.58749	25.5232	0.643083	31.89515	40.30933
Pg140	73.72299	72.46381	49.46903	35	42.41974	Hg153	492.3627	577.014	925.105	1397.152	354.035
Pg141	162.5896	151.2043	146.9895	135.9768	147.785	Hg154	34.75965	33.51306	59.69694	60	59.91993
Pg142	71.31964	67.96666	64.26362	125.8	65.17869	Hg155	16.07438	51.50848	59.94694	60	59.83564
Pg143	171.6939	110.9543	111.3473	233.3459	83.79595	Hg156	63.97824	70.72145	0.902541	120	119.5585
Pg144	74.93626	84.43365	48.01269	44.00456	75.66889	Hg157	79.19911	49.06161	2.204878	0	119.9105
Pg145	42.10098	34.04034	38.92032	60	10.26045	Hg158	620.9072	685.6878	909.6608	0	356.0761
Pg146	78.11662	81.77873	35.49127	105	50.79323	Hg159	23.35992	36.49364	0.132797	60	59.7823
Pg147	138.7505	181.9151	134.0777	152.7939	153.2985	Hg160	42.2545	21.84263	58.04509	60	59.93881
Pg148	98.76258	67.52022	106.5738	125.8	92.43806	Hg161	65.53427	85.31346	119.9548	120	119.6182
Pg149	123.4756	138.6376	120.5755	246.9105	84.26077	Hg162	88.75652	59.12272	119.9832	120	32.51809
Pg150	100.8023	92.11579	57.43339	43.09641	81.89629	Hg163	593.1704	557.3912	910.0227	1552.225	451.6828
Pg151	47.5644	49.11082	39.03834	21.99167	39.08587	Hg164	40.84546	28.39691	6.243836	0	59.93934
Pg152	71.07851	73.09166	35.52999	62.7455	79.6928	Hg165	26.72567	40.88351	1.255413	0	59.82737
Hg105	143.0187	142.1079	177.43	135.7857	108.7822	Hg166	59.93746	67.65058	0.096145	0	119.9096
Hg106	90.88735	79.54195	130.1675	0	92.10741	Hg167	108.2142	103.2702	0.040991	120	119.8066
Hg107	126.5657	129.3163	0.000412	132.2196	123.4405	Hg168	651.7794	522.4332	906.7537	0	363.8524
Hg108	69.20408	94.21251	109.3067	92.84554	83.27392	Hg169	10.41676	45.48739	6.791869	60	59.87452
Hg109	31.29471	25.45084	0	49.28014	40.97655	Hg170	13.80173	32.38762	1.068761	60	59.89786
Hg110	30.37513	22.48432	0.134585	41.0851	30.57175	Hg171	87.94417	77.85746	8.44×10^−05^	120	117.9236
Hg111	103.5517	115.8397	0.92819	9.79×10^−05^	165.9765	Hg172	86.86305	55.04469	5.058568	120	119.6775
Hg112	97.67907	47.03794	9.108935	7.216803	120.0454	Hg173	737.661	585.6358	920.5892	0	357.0134
Hg113	127.911	152.0281	178.3112	135.6339	132.3767	Hg174	7.250124	18.07854	0.274126	60	59.97047
Hg114	78.25294	66.01586	0.887772	4.642805	100.8766	Hg175	46.82325	26.62512	0.685941	60	59.89434
Hg115	39.48022	28.2455	0.111545	0	41.79333	Hg176	104.8884	84.52888	20.36861	0	119.7378
Hg116	16.99868	41.49891	27.29432	0	26.38251	Hg177	59.65188	72.10567	119.9272	120	114.6435
Hg117	88.30371	100.3173	174.6643	177.7991	124.3396	Hg178	716.495	602.1813	907.8548	0	387.0579
Hg118	56.75794	55.3174	0.059311	0	84.03871	Hg179	50.7917	27.42597	3.635189	60	59.92259
Hg119	136.3953	134.5814	3.576152	176.4035	139.0128	Hg180	25.99096	59.86144	13.01303	60	59.87757
Hg120	94.99244	102.3033	0.2653	0	122.1011	Hg181	106.4563	119.7318	0.001621	120	119.9104
Hg121	13.33646	25.92046	0.414366	43.82525	40.565	Hg182	43.1831	47.23645	0.604342	120	119.8964
Hg122	27.42141	24.99265	1.737371	0.141283	21.96434	Hg183	528.5626	765.2542	907.7043	1465.766	354.6583
Hg123	63.13502	112.5775	163.3978	0	137.3244	Hg184	41.81199	47.29115	0.444441	0	59.7352
Hg124	35.0343	56.9659	87.60028	31.82819	103.3148	Hg185	48.15765	32.108	2.205178	60	36.09575
Hg125	73.4311	59.39658	0.75323	0	122.4838	Hg186	49.18479	97.81689	0.038816	120	119.7626
Hg126	57.58084	90.97783	1.747817	0	93.08094	Hg187	88.52079	40.21094	0	120	114.5619
Hg127	7.690224	25.85509	0.069185	0	51.25276	Hg188	739.6827	757.5151	899.629	1401.145	400.6007
Hg128	20.55402	26.5521	0.001147	30.13535	25.26957	Hg189	50.24342	38.4695	59.96731	60	23.89247
Hg129	99.64527	113.7313	167.7151	132.7904	121.0729	Hg190	34.05605	26.36133	59.97419	0	59.967
Hg130	66.35591	87.23498	0	0	85.29981	Hg191	106.9444	119.9435	119.9653	0	119.9612
Hg131	141.8201	58.84182	0.001814	153.311	162.7133	Hg192	57.37515	75.64223	120	120	119.7678
Hg132	102.1795	59.0631	0.032602	118.7186	54.42837	Sum (Pg)	18,800	18,800	18,800	18,800	18,800
Hg133	22.77924	16.49257	7.884998	0	43.44979	Sum (Hg)	10,000	10,000	10,000	10,000	10,000
						WCTF ($)	581,798	572,324.8	678,051.9	793,224.8	487,145.2
